# Dr. Griffin’s Case of Complicated Fractures of Both Thighs, and of the Upper and Lower Jaws, with Diastasis of the Former

**Published:** 1842-05-14

**Authors:** Richard Griffin

**Affiliations:** Norwich


[We give the following article entire, although somewhat carelessly reported, as it is well calculated to impress upon surgeons of slender experience the propriety of attending with care to the minute details of surgical treatment, even under the most unfavourable circumstances. Had the position of the teeth been neglected by Mr. Griffin in this case, or had this gentleman removed the fragments of bone which were entirely detached from the jaws, and, in the lower jaw, almost separated from the soft parts also, his patient would have recovered with very serious deformity. Even in uncomplicated compound fractures it is too fashionable to tamper with loose fragments, most of which will reunite, if the periosteum remains freely attached to the surrounding soft parts, though no considerable osseous arteries may enter them; and the usefulness of many a limb has been sacrificed by pseudarthrosis produced by removing detached pieces of bone under the influence of the ridiculous hyper-refinement of the physiological theorists of certain schools, who endeavour to attribute distinct functions to every little arterial branch of special destination, regardless of the vicarious power by which almost any organ may assist in fulfilling the office of another when severe necessity demands the exertion. We fear that most surgeons would have sacrificed or neglected the central fragment of the lower jaw in this case, either from error of doctrine, or from the idea that the patient would necessarily die. Extensive fractures of the face are usually the result of tremendous forces, and very generally terminate fatally.]

*Case of Complicated Fractures of both Thighs, and of the Upper and Lower Jaws, with Diastasis of the former.* By Richard Griffin, M. R. C. S., of Norwich.—Captain T., ætat 28, was driving a phaeton down a steep hill, when the horse took fright, and, coming in contact with a miller's waggon, dashed the carriage to pieces, and he received the following injuries:—An oblique fracture of the left thigh, near the knee-joint, and a transverse one of the right thigh, about its centre. For these I placed him on a double-inclined bedstead, and treated him in the usual way with perfect success. There were two wounds near the chin, each about two inches long, which required sutures to keep them in their proper position; and union took place by granulations in the course of a fortnight, leaving, at the end of three months, only faint lines indicating their former situation. The foreign body, causing one of these wounds, penetrated into the mouth, and cut through the lower jaw, immediately beneath the incisor and canine teeth, completely separating a horizontal portion of it containing these teeth, the only connecting medium left being a part of the gum in the inside. A medical gentleman present, considering its re-union improbable, proposed that the slight attachment should be cut through and the part removed; but this I declined, believing no harm would ensue in attempting to save it, though incalculable good would result, should it be successful. I therefore brought the part nearly into its natural position, and retained it there by wires fastened round the teeth contained in it, and to the adjoining fixed teeth. I found it impossible to get them to the same level as the others, the unnatural elevation being about one-eighth of an inch; but, in the course of three weeks, had the pleasure of observing them become gradually drawn down to their natural situation, by contraction of the granulations. The entire gum on the outside sloughed away, and caused the mouth to be very offensive, requiring chloride of lime gargles. At this time I doubted whether I should be enabled to save it; but after three days, granulations began to spring up from the surface of the bone, and covered it. These I had some trouble to prevent adhering to the wound in the lip, which was avoided by keeping lint constantly inserted between them. At the end of three months no ossific union had taken place, the bond being apparently ligamentous, thus permitting a slight motion. In every other respect the teeth were nearly as useful as before, and showed no disfigurement, or traces of so severe an accident. The superior maxilla of the right side was loose, having been separated at the sutures; so that by taking hold of the teeth inserted into it between the finger and thumb, the entire maxilla could be moved; this, in three or four weeks, became firmly fixed, not having required any treatment. The posterior part of this maxilla, containing the three last teeth, was also fractured, the teeth, with the bone attached, dropping a quarter of an inch below those adjoining. I fixed them with wire to the firm teeth; bnt they, like those in the inferior maxilla, did not unite by bone, although they became tolerably firm, and allowed only a slight degree of motion. This fracture gave me considerable trouble, not only in fixing the teeth, which were so close together that the wire could only be passed between their necks, and required much perseverance to twist it effectually, but from two abscesses which formed, and continued to discharge for two months, one internally and the other externally, through the cheek. From the latter several pieces of bone came away; and they ultimately healed, followed by the skin itself becoming attached to the bone, leaving a permanent depression, or dimple, in the cheek. Some of the front teeth of the upper jaw were also loosened, and an abscess formed connected with them, which discharged for some weeks, and several pieces of bone were detached. The zygomatic process of the right os malæ was fractured, and nature there perfected the cure, leaving a slight inequality only to show the seat of the injury. Soon after the patient was placed in bed he three times vomited blood of a dark colour, to the extent of nearly two pints, mixed, in all probability, with fluids of which he had partaken during the day. This was followed by moaning and a frightful state of collapse, the wrist being almost pulseless, and he appeared to be rapidly sinking. I immediately gave him 60 drops of laudanum in a small quantity of brandy and water, which I repeated three times in the course of two hours, when he began to rally, and he has since told me that each dose appeared to give him "fresh life." This state of collapse was brought on by the severity of the accident, conjoined to exposure in an open cart for some miles, on a very cold night. The blood vomited arose probably from the wounds in the mouth, and was swallowed, and not from injury of an internal part; had the latter been the cause, there would have been some marks of violence, or at least pain, denoting the injured part. This case is interesting, as it teaches us that wounds will frequently unite which are considered almost hopeless. I saw it still more strongly exemplified, some years since, at the Norfolk and Norwich Hospital, in a child whose leg was nearly cut off by a waggon passing over it; indeed, not more than a third of the muscle and integument remained that were not severed, the bones being both fractured. The late Mr. Martineau, whose case it was, immediately decided upon trying to save it, contrary to the opinion of all present, remarking, that as the patient was young and in good health, nature might effect a cure. And it was worth the attempt. In this case he was correct, as the child perfectly recovered.—*Ibid.*

